# Joint recording of EEG and audio signals in hyperscanning and pseudo-hyperscanning experiments

**DOI:** 10.1016/j.mex.2021.101347

**Published:** 2021-04-20

**Authors:** Alejandro Pérez, Philip J. Monahan, Matthew A. Lambon Ralph

**Affiliations:** a MRC Cognition and Brain Sciences Unit, University of Cambridge, United Kingdom; b Department of Language Studies, University of Toronto Scarborough, Canada; c Department of Psychology, University of Toronto Scarborough, Canada; d Department of Linguistics, University of Toronto, Canada

**Keywords:** Inter-brain, Brain-to-brain, B2B, 2PN

## Abstract

Hyperscanning is an emerging technique that allows for the study of brain similarities between interacting individuals. This methodology has powerful implications for understanding the neural basis of joint actions, such as conversation; however, it also demands precise time-locking between the different brain recordings and sensory stimulation. Such precise timing, nevertheless, is often difficult to achieve. Recording auditory stimuli jointly with the ongoing high temporal resolution neurophysiological signal presents an effective way to control timing asynchronies offline between the digital trigger sent by the stimulation program and the actual onset of the auditory stimulus delivered to participants via speakers/headphones. This configuration is particularly challenging in hyperscanning setups due to the general increased complexity of the methodology. In other designs using the related technique of pseudo-hyperscanning, combined brain-auditory recordings are also a highly desirable feature, since reliable offline synchronization can be performed by using the shared audio signal. Here, we describe two hardware configurations wherein the real-time delivered auditory stimulus is recorded jointly with ongoing electroencephalographic (EEG) recordings. Specifically, we describe and provide customized implementations for joint EEG-audio recording in hyperscanning and pseudo-hyperscanning paradigms using hardware and software from Brain Products GmbH.

• Joint EEG-audio recording configuration for hyperscanning and pseudo-hyperscanning paradigms.
• Near zero-latency playback of auditory signal captured by a microphone.
• Precise alignment between EEG and auditory stimulation.

## Graphical abstract

**Figure:**
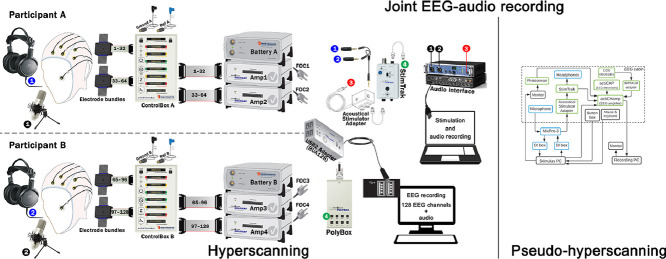

**Table:** Specifications table

Subject Area:	Neuroscience
More specific subject area:	*Electrophysiology*
Method name:	*EEG-audio hyperscanning and pseudo-hyperscanning*
Name and reference of original method:	*NA*
Resource availability:	*NA*

## Method details

### Methodological background

Hyperscanning refers to obtaining simultaneous neural recordings from more than one person [11]. Like hyperscanning, pseudo-hyperscanning also demands synchronization between recordings. The principal difference between the methodologies is that pseudo-hyperscanning requires the alignment of signals recorded from different brains obtained at different times. Both techniques are commonly used inside the ‘two-person neuroscience’ (2PN) framework [7] to investigate the specific neural mechanisms underpinning social interaction. Results revealed that effective communication is associated with a pattern of inter-personal neural covariation. Hyperscanning using EEG is becoming increasingly popular, since it allows researchers to explore the interactive brain with high temporal resolution; however, to date, no specific EEG hyperscanning devices have been developed.

To address this issue, some effort has been made in describing EEG hyperscanning configurations with sufficient detail to be easily reproduced by others [2,10]. With increased use, methodologies related to hyperscanning recordings are improving their best practice standards [1]. In addition to the perfectly synchronized and independent neural recordings, there is also the expectation to include and analyze exogenous sensory signals (e.g., audio and luminance). The inclusion of these signals helps to better characterize the complexity of the dynamical mutual interacting system that is established during a joint action. Thus, besides the technological challenges of the EEG hyperscanning implementation, researchers have provided different solutions for the synchronized recording of ‘external’ signals and EEG hyperscanning. Specific efforts for incorporating synchronized auditory signals include the use of software for hardware interfacing, both open-source (e.g., Lab Streaming Layer, LSL) [6] and commercial [5]; audio fed to amplifier [13], as well as hardware solutions for voice onset detection and marking [12]. In the case of EEG pseudo-hyperscanning designs using auditory stimuli, combined brain-auditory recordings is also desirable, since it allows for exact off-line synchronization by using the shared auditory signal. Similar to EEG hyperscanning, among other solutions, the auditory signal has been fed to the amplifier in EEG pseudo-hyperscanning experiments [9]. Here the aim is to offer a step-by-step description of solutions to synchronous audio-EEG recordings. Specifically, we describe and provide customized implementations for joint EEG-audio recording in hyperscanning and pseudo-hyperscanning setups using hardware and software from Brain Products GmbH.

## Methodological details

Measuring auditory time-locked neurophysiological responses requires precise temporal synchrony between the delivery of the auditory signal to the participant and the sending of the digital trigger (marker) to the acquisition device. Often, due to imprecision in hardware and software, an asynchrony is observed between the stimulus and digital trigger [4]. If these timings are incorrectly marked in the neurophysiological signal, it is near impossible to correctly align the brain responses with the sensory stimulus, reducing the precision and quality of our analyses. For example, in the investigation of fast brain dynamics associated with tracking a rapidly evolving auditory signal, we rely on the robustness of the marker timing to capture speech envelope tracking. Any errors in timing precision will cause a blurring between the brain-audio coupling measured on different conditions (or between real data and surrogate data). In other words, markers that are more reliable yield a reduced Type II error rate when comparing between experimental conditions.

When possible, it is advisable to use hardware synchronization to control for timing delays. Here, we advocate recording the auditory signal as an additional signal directly incorporated into the neurophysiological recording. This presents an effective way to control for offline any time shift between the digital trigger sent by the stimulation program and the actual onset of the auditory stimulus delivered to participants via speakers/headphones. Afterward, marker timing can be adjusted to correctly synchronize with the auditory stimulus. Computing the cross–correlation function between the auditory signal recorded within the neurophysiological recording and the corresponding audio file is one possible offline method to correct for any delays. The inclusion of the auditory signal is straightforward in magnetoencephalographic (MEG) recordings, since the system by default includes dedicated channels to record experimental stimulation; however, it is less common in EEG recordings, where the implementation of additional hardware connections is required. This configuration is particularly challenging in hyperscanning setups due to the already increased complexity of the methodology. While not necessary for the implementation of hyperscanning or pseudo-hyperscanning experiments, including the auditory signal within the neurophysiological recording is desirable, as it more easily allows researchers to deal with potential timing issues. The following are the solutions to the implementation of joint audio-EEG recordings.

## Hyperscanning

The hyperscanning set-up presented here describes the configuration for 64-channel simultaneous EEG recordings of two individuals. The number of channels can, of course, be expanded or reduced.

### Hardware

• Microphone with cable. 2 units
• Audio interface. 1 unit
• Headphones. 2 units
• Headphone splitter. 1 unit
• actiCAP EEG caps. 2 units
• actiCAP 32 channel EEG electrode set. 4 units
• Reference (REF) and Ground (GND) electrodes. 2 units
• actiCAP ControlBox and USB cable. 2 units
• AA NiMH batteries (capacity>=2100 mA/h). 10 units
• Flat ribbon cable. 2 units
• BrainAmp Standard amplifier with battery connection cable. 4 units
• PowerPack rechargeable battery. 2 units
• Fiberoptic cable. 4 units
• USB2 Adapter (BUA128) and USB cable. 1 unit
• Computer (Recording and Stimulation). 2 units
• Acoustical Stimulator Adapter. 1 unit
• StimTrak device including cables. 1 unit
• Auditory adaptor for StimTrak
• PolyBox. 1 unit

### Software

• BrainVision Recorder
• actiCAP ControlSoftware
• Software for controlling the audio interface
• Software for stimulation and audio recording (e.g., PsychoPy)

### Dual-EEG setting

For each participant in a dyad, electrophysiological data is acquired with a 64-channel actiCAP active electrode array, which is created with two electrode bundles or two electrodes’ branch. The electrodes are mounted on an actiCAP elastic cap with a layout according to the International 10–20 system. Both actiCAP bundles, jointly with the Reference (REF) and Ground (GND) electrodes, are connected to one actiCAP ControlBox. In turn, the ControlBox is connected using flat ribbon cables to two amplifiers BrainAmp Standard. A PowerPack rechargeable battery feeds the amplifiers. Different participants use different PowerPack batteries to avoid a galvanic bridge. The amplifiers send the EEG signal to the USB2 Adapter (BUA128) via Fiberoptic cables (FOC), which digitize and send the data to the recording computer via a USB cable.

The two participants connect differently to the actiCAP ControlBox. One participant (henceforth, participant A) connects to the splitter box sockets and amplifier sockets corresponding to channels 1–32 (green) and 33–64 (yellow). Amplifier socket 1–32 connects to an amplifier that we will term ‘amplifier one’. This should be connected to the port labelled Fiberoptic 1 of the BUA128. Amplifier socket 33–64 connects to ‘amplifier two’, which should be connected to the port Fiberoptic 2 of the BUA128. For the second participant (henceforth, participant B), channel assignment begins where participant A ends. Participant B connects to the splitter box sockets and amplifier sockets corresponding to channels 65–96 (red) and 97–128 (white). Amplifier socket 65–96 connects to ‘amplifier three’, connected to Fiberoptic 3. Amplifier socket 97–128 connects to ‘amplifier four’, connected to the port Fiberoptic 4. Note that recordings from the two participants co-occur at the USB2 Adapter (BUA128) and connect to the Recording computer where signals are synchronously recorded as 128 EEG channels by using the BrainVision Recorder software.

The markers (triggers) sent by the Stimulation PC pass through the trigger cable connecting to the 26-pin HD d-Sub socket (Trigger port) of the USB2 adapter (BUA); however, one might want to use a laptop for stimulation where a parallel port is not available. A possible solution is to send triggers using a USB port to a TriggerBox that connects to the BUA.

### Impedance measurement

The actiCAP ControlSoftware is used to measure impedances. The software can be used directly or within the BrainVision Recorder interface. Impedance measurements should be conducted one participant at the time and using a workspace template for 64 channels, including amplifiers ‘one’ and ‘two’. The amplifiers ‘three’ and ‘four’ should be turned off. The electrode bundles for each participant should be plugged/unplugged to the splitter box sockets and amplifier sockets corresponding to channels 1–32 (green) and 33–64 (yellow), including their own REF and GND. It is recommended to restart the actiCAP ControlSoftware before each measurement. To ensure a stable power supply during impedance measurement, power should only be supplied to the actiCAP ControlBox via a USB port. Specifically, this can be achieved with an active USB hub with a separate power supply on your computer (i.e., powered USB hub). Then, switch to the AA batteries when you are recording EEG data.

### Audio settings

The Fireface UCX audio interface is connected with a USB cable to the stimulation PC and it is controlled by the software TotalMixFX (v1.61). These controls include, among others, routing of the signal, providing phantom power to the microphones, the headphone mix, the mono/stereo mode and the gain. A workspace file including the TotalMixFX software configuration is provided as Supplementary Material 1.

Input audio is received at two condenser microphones TM-80 (TASCAM) that are connected to the Mic/Line of a Fireface UCX audio interface (RME) using XLR cables. Input audio is delivered (loopback) from the Phones output TRS jack of the Fireface UCX to the Acoustical Stimulator Adapter (Brain Products GmbH). Then, the signal is delivered twofold, to a headphone splitter (Syncwire) connecting to two headphones HA RX700 (JVC) and through the StimTrak device to the PolyBox. Finally, the PolyBox connects to the “AUX” port of the BUA128.

Thus, signals recorded in BrainVision Recorder include the 128-channel EEG signals from both participants and one external channel with an auditory signal delivered by the headphones. The BrainVision Recorder workspace is provided as Supplementary Material 2.

### Hints

Polybox has a hardware cut-off of 100 Hz. This removes high frequency components of the auditory signal.

PolyBox is no longer available or in production by Brain Products GmbH.

## Pseudo-hyperscanning

Pseudo-hyperscanning is defined as the alignment of signals recorded from different brains at different times by using as reference a physical signal common to the different recordings. This is commonly accomplished by using a marker. The pseudo-hyperscanning set-up presented here describes the configuration for 32-channel EEG recordings. The number of channels can, of course, be expanded or reduced.

### Hardware

• actiCAP 32 channel EEG electrode set. 1 units
• actiCAP EEG caps. 1 unit
• Reference (REF) and Ground (GND) electrodes. 1 unit
• electrooculography (EOG) electrodes. 4 units
• BIP2AUX adapters. 2 units
• actiCHamp EEG amplifier. 1 unit
• actiPOWER battery pack. 1 unit
• StimTrak device including cables and two AA batteries. 1 unit
• Acoustical Stimulator Adapter. 1 unit
• Photosensor. 1 unit
• Direct Injection (DI) box. 2 units
• Mixer/recorder device. 1 unit
• Headphones. 1 unit
• Microphone. 1 unit
• Stimulus and recording PC. 1 unit each
• Button box. 1 unit

### Software

• BrainVision Recorder software
• Software for stimulation and audio recording (e.g., PsychoPy)

### EEG settings

Electrophysiological data are acquired with a 32-channel actiCAP active electrode array mounted in an actiCAP elastic cap, and a one-module actiCHamp amplifier. The amplifier is fed by an actiPOWER battery pack. Horizontal and vertical eye movements are monitored using four additional electrooculography (EOG) electrodes connected to two BIP2AUX adapters and then to two different AUX (auxiliary) inputs of the actiCHamp. A StimTrak device and a photosensor attached to the screen monitor are also connected to AUX inputs. The StimTrak received the auditory signal via the pass-through connector. Thus, signals recorded in the actiCHamp amplifier include: the EEG signal, any auditory signal delivered by the headphones and the photosensor's signal. EEG-audio-photosensor signals are jointly recorded on the same workspace using the Recorder software.

### Audio settings

Auditory stimuli are delivered from the Stimulation PC through the soundcard's headphone output to one direct injection (DI) box via a cable with TRS connectors. The DI box is then connected to Panel 1 of a MixPre-3 mixer/recorder device with an XLR cable. The MixPre-3 deliver the auditory signal from the Headphone Output by connecting to the Acoustical Stimulator Adapter part of the StimTrak device. Finally, the signal is delivered twofold, through the StimTrak to the amplifier and to the Beyer Dynamic DT 770 PRO over-ear headphones used for presenting auditory stimuli. In turn, speech is recorded with a AT 3035 Cardioid microphone placed in front of the participant. The mic is connected to Panel 3 of the MixPre-3 using an XLR cable. The MixPre-3 delivers the signal from the Stereo-Out panel to a second DI box using a cable with TRS jack connectors. Finally, the DI box is connected using TRS jack connectors to the soundcard's analogue input (Line-in/Mic-in) at the Stimulation PC. The two DI boxes with ground lift are added to avoid ground loops between the Stimulation PC and the Mix-Pre 3. Both signals, from the microphone and the Stimulation PC, are mixed in the MixPre-3 and sent to the headphones. Therefore, the headphones (loopback) play the sound detected by the microphone. For each participant, the gain of the auditory signals is semi-automatically adjusted to match the intensities of the immediate playback and the presentation of the recorded sounds by using a custom-made program.

### Hints

StimTrak has a hardware bandwidth of 0–10 kHz. Therefore, while higher frequencies (> 10 kHz) can be recorded with the actiCHamp amplifier, StimTrak is only able to sample the auditory signal at the lower high-frequency cut-off.

Off-line, it is possible to remove any timing latency offsets between when the digital trigger is sent by the stimulation program and the actual playback of the auditory stimulus. This can be achieved by computing the cross⁠–correlation function between the auditory signal recorded with the EEG and the audio file.

## Additional information

**Fig. 1:**
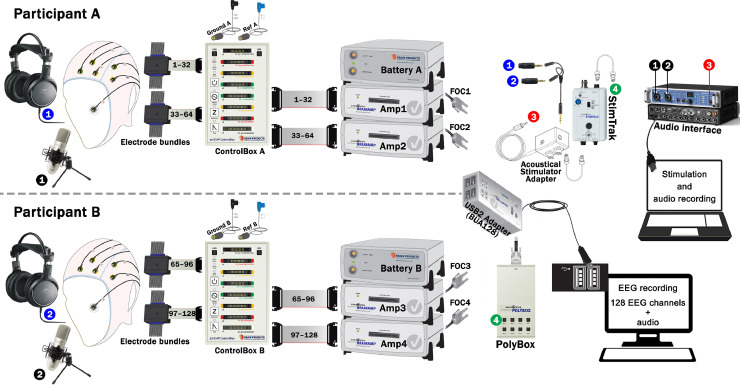
Configuration of the EEG-audio system in the hyperscanning setup. Critical elements and its connections are included. Circles with the same number and color indicate a connection.

**Fig. 2:**
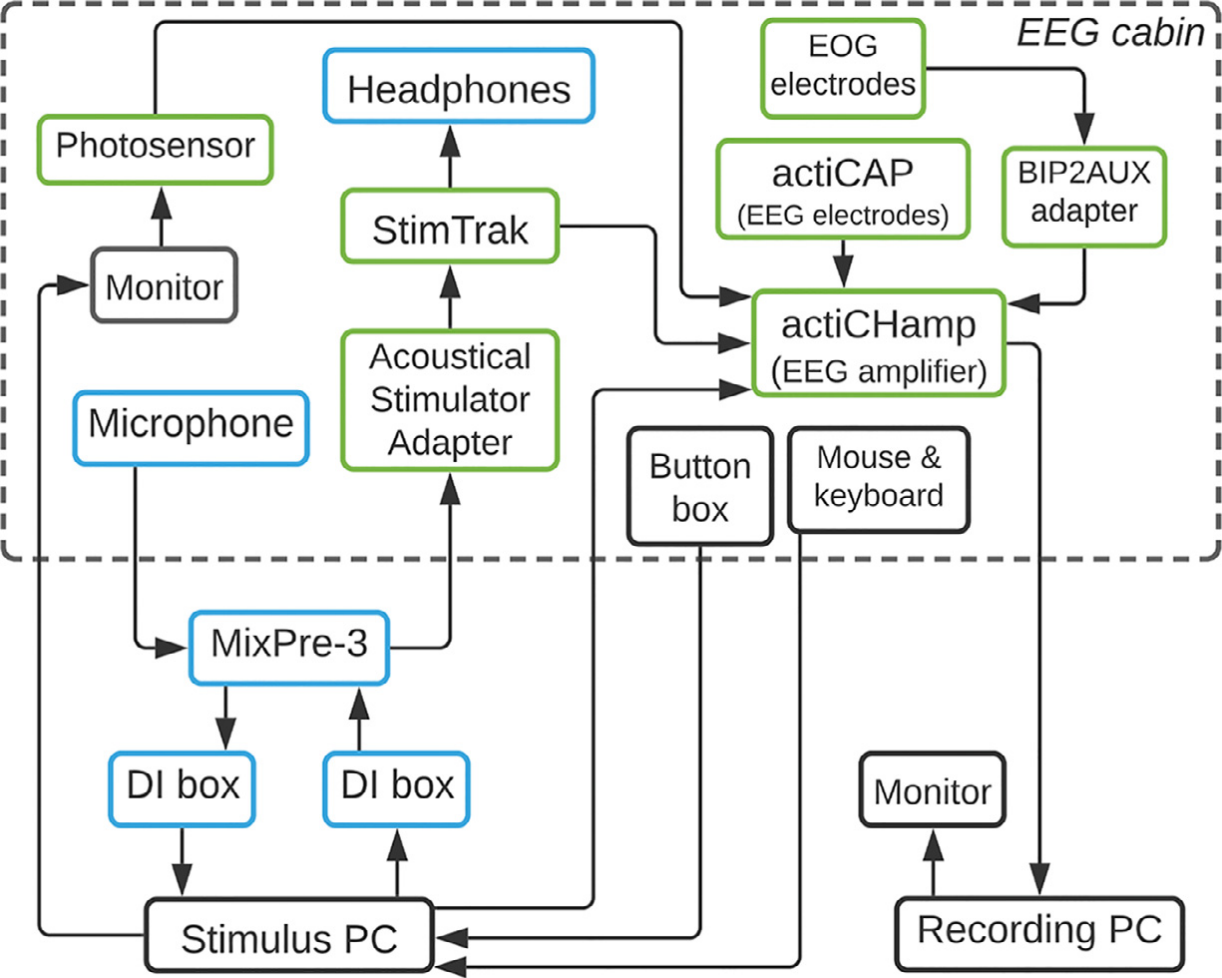
Diagram of the physical configuration of the EEG-audio system in the pseudo-hyperscanning setup, including hardware elements, connections and the direction of signal flow. Each box with a name represents an individual device: EEG-related devices are in green, audio-related devices are in blue, computer-related devices and other peripherals are in black. Solid lines connecting boxes represent cable connections between devices. The arrow indicates the direction of signal flow. The dashed-rectangle represents the EEG (sound-attenuated) cabin.

Other EEG manufacturers offer equivalent adapters for the collection of hardware synchronized auditory signals. Therefore, similar solutions to the ones offered here could be implemented in different EEG systems. In addition, there are open-source alternatives to the proprietary adapters that offer a cost-effective solution. For example, the EEG Synchronization Box (ESB) allows for hardware-based synchronization [3]. ESB ensures reliable EEG timing with stimulus onsets and other devices. Moreover, some microcontrollers, such as Arduino or Raspberry Pi Pico, allow creating similar devices at a much lower cost, sometimes even including EEG acquisition [8] (Figs. 1, 2).

## Declaration of Competing Interest

The authors declare that they have no known competing financial interests or personal relationships that could have appeared to influence the work reported in this paper.
